# Supplementary material for: Saccharomyces boulardii (CNCM I-745) improves intestinal damage in sepsis by remodeling intestinal flora structure
Source: Front Cell Infect Microbiol. 2025 Jul 31;15:1602792. doi: 10.3389/fcimb.2025.1602792 (PMC12350329; doi:10.3389/fcimb.2025.1602792)
Supplement: Supplementary file 1 [file DataSheet1.zip › summary/summary/7_Differential_analysis/2_LEfSe_result/Group_DW4_vs_DW0_vs_CW4_vs_CW0_vs_BW4_vs_BW0_vs_AW4_vs_AW0_0.05_3.0/LEfSe_Group_DW4_vs_DW0_vs_CW4_vs_CW0_vs_BW4_vs_BW0_vs_AW4_vs_AW0.g.barplot.pdf]

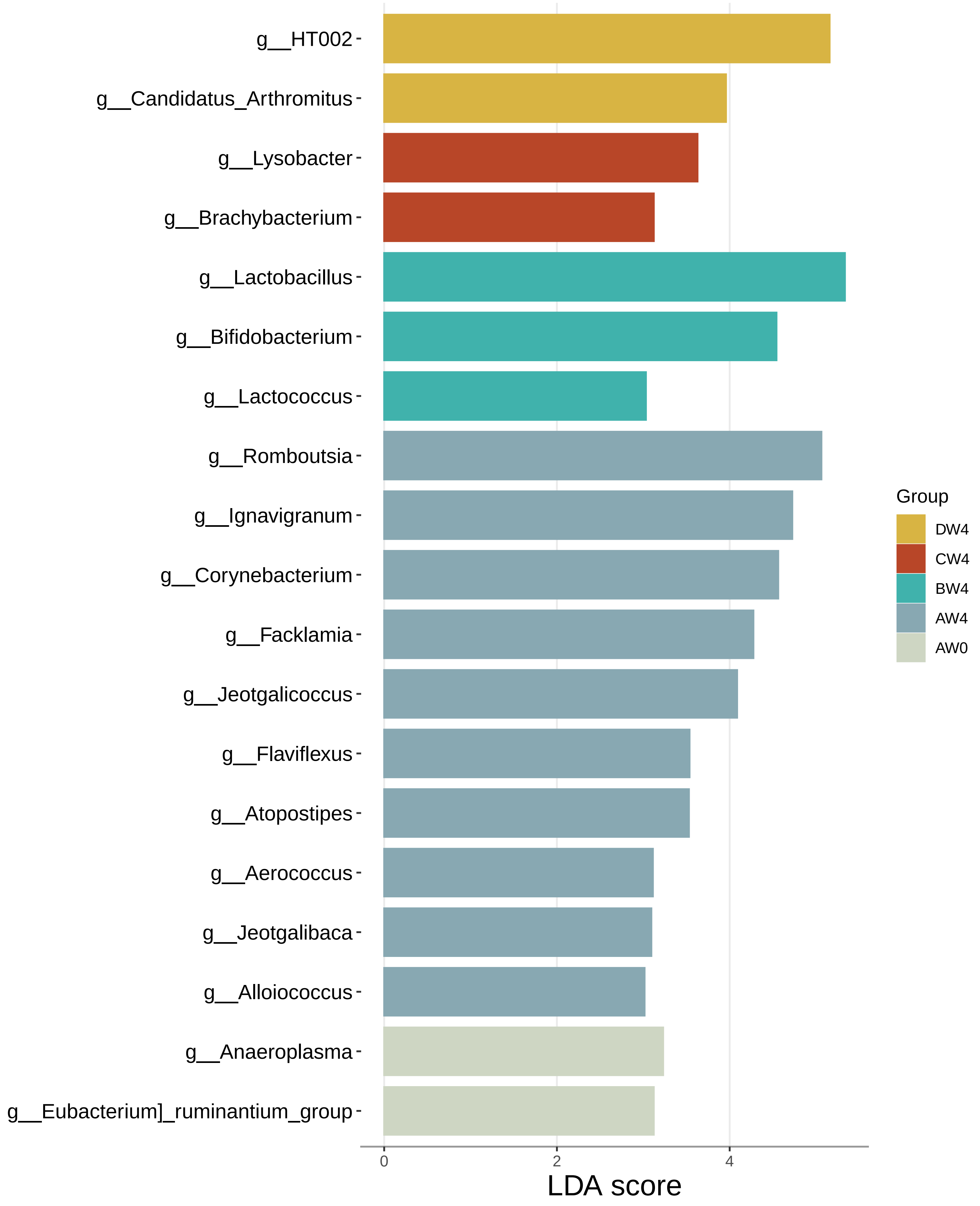
g__HT002
g__Candidatus_Arthromitus
g__Lysobacter
g__Brachybacterium
g__Lactobacillus
g__Bifidobacterium
g__Lactococcus
g__Romboutsia
g__Ignavigranum
g__Corynebacterium
g__Facklamia
g__Jeotgalicoccus
g__Flaviflexus
g__Atopostipes
g__Aerococcus
g__Jeotgalibaca
g__Alloiococcus
g__Anaeroplasma
g__Eubacterium]_ruminantium_group
0
2
4
LDA score
Group
DW4
CW4
BW4
AW4
AW0
